# Stereotactic body radiotherapy plus lenvatinib and sintilimab with and without transarterial embolization for advanced hepatocellular carcinoma with portal vein tumor thrombus: a dual-center, propensity score-matched retrospective analysis

**DOI:** 10.3389/fimmu.2026.1644027

**Published:** 2026-03-04

**Authors:** Jun Jia, Cong Ning, Quan Wang, Jing Sun, Xinmu Zhang, Taifeng Zhu, Duo Li, Haitao Zhao, Xuezhang Duan

**Affiliations:** 1 Department of Radiotherapy, Senior Department of Oncology, Chinese PLA General Hospital, Chinese PLA Medical School, Beijing, China; 2 Department of Liver Surgery, State Key Laboratory of Complex Severe and Rare Diseases, Peking Union Medical College Hospital, Chinese Academy of Medical Sciences and Peking Union Medical College (CAMS & PUMC), Beijing, China; 3 Department of Clinical Nutrition & Health Medicine, State Key Laboratory of Complex Severe and Rare Diseases, Peking Union Medical College Hospital, Chinese Academy of Medical Sciences & Peking Union Medical College (CAMS & PUMC), Beijing, China

**Keywords:** hepatocellular carcinoma, immunotherapy, locoregional therapy, stereotactic body radiotherapy, transarterial embolization

## Abstract

**Introduction:**

Portal vein tumor thrombus (PVTT) in hepatocellular carcinoma (HCC) is associated with poor prognosis and limited efficacy of current first-line therapies. Combining locoregional and systemic therapies may enhance antitumor immunity. However, the safety and efficacy of dual locoregional therapy (LRT) with stereotactic body radiotherapy (SBRT) and transarterial embolization (TAE) along with targeted immunotherapy is unclear.

**Methods:**

In this retrospective real-world study, we analyzed 204 patients with Barcelona Clinic Liver Cancer stage C HCC and PVTT treated with SBRT plus lenvatinib and sintilimab, with or without TAE, between June 2018 and December 2022. Propensity score matching (PSM) was performed to balance baseline characteristics. The primary endpoints were progression-free survival (PFS) and overall survival (OS), and the secondary endpoints included local control (LC) and safety.

**Results:**

After PSM (64 patients per group), the TAE group showed significantly longer median PFS (11.0 vs. 6.0 months; HR = 0.71, p=0.044) and a trend toward improved LC than the non-TAE group (51.0 vs. 36.0 months; HR = 0.54, p=0.066), but comparable OS (19.0 vs. 18.0 months; p=0.606). Multivariate analysis confirmed TAE as an independent predictor of reduced risk of progression (HR = 0.52, 95% CI: 0.36–0.76). Objective response rates (40.6% vs. 39.1%, p=0.861) and grade ≥3 treatment-related adverse events (50.0% vs. 50.0%, p=0.854) were similar between groups. TAE did not increase hematologic or hepatic toxicity, supporting its tolerability.

**Discussion:**

Adding TAE to SBRT, lenvatinib, and sintilimab prolonged the median PFS in patients with advanced HCC and PVTT, showing comparable safety. This real-world study supports dual LRT combined with targeted immunotherapy as a feasible treatment option and merits prospective validation.

## 1 Introduction

Portal vein tumor thrombus (PVTT), a common complication of hepatocellular carcinoma (HCC), has an incidence of 44.0–66.2%, suggesting a poor prognosis (1–3). Given PVTT’s association with portal hypertension, liver dysfunction, and tumor invasion, previous studies have shown that first-line treatment for advanced HCC (i.e., atezolizumab plus bevacizumab) has a 23% objective response rate (ORR) and median overall survival (OS) of 7.6 months for a subgroup of patients with HCC with PVTT (4, 5). Consequently, new strategies are needed to improve the efficacy of targeted immunotherapies.

Commonly used locoregional therapies (LRT), including radiotherapy or intra-arterial therapy, directly kill tumor cells and release an abundance of tumor antigens that could effectively elicit systemic antitumor immune responses (6–8). However, the efficacy of this antigen release is frequently constrained by the pre-existing immunosuppressive tumor microenvironment (TME) (9). In this context, sintilimab, a PD-1 inhibitor, acts to block the PD-1/PD-L1 checkpoint, thereby reversing T-cell exhaustion and potentiating their anti-tumor cytotoxicity (10). Meanwhile, the multi-kinase inhibitor lenvatinib contributes by normalizing the aberrant tumor vasculature, a process that alleviates hypoxia and can remodel the immunosuppressive TME, thereby facilitating enhanced infiltration and function of immune effector cells (11). This provides a strong rationale for the combined application of LRT and systemic therapy in advanced HCC (12).

Although combined radiotherapy and transarterial (chemo)embolization (TA(C)E) or hepatic arterial infusion chemotherapy can provide a higher tumor response and better survival benefits than monotherapy for patients with HCC showing macrovascular invasion, the increased risk of adverse events (AE) caused by dual LRT poses a safety challenge to its application (13–16). Thus, no clinical study has been conducted on combining dual LRT and targeted immunotherapy in patients with advanced HCC. The aim of this study was to compare the efficacy and safety of stereotactic body radiotherapy (SBRT) plus lenvatinib and sintilimab with TAE versus SBRT plus lenvatinib and sintilimab triple therapy as a first-line treatment in patients with HCC with PVTT.

## 2 Materials and methods

### 2.1 Patient data

This retrospective study was conducted using data from patients with HCC and PVTT who were administered SBRT plus lenvatinib and sintilimab, with or without TAE, at Hospital A and Hospital B between June 2018 and December 2022. This study was approved by our Institutional Review Board. The requirement for informed consent was waived owing to the retrospective nature of the study. The study inclusion criteria were as follows: (1) histologically or radiographically confirmed HCC with PVTT; (2) treatment with lenvatinib and sintilimab as the first-line systemic therapy following SBRT; (3) interval between SBRT and TAE ≤ 2 weeks; (4) age ≥18 years; (5) Eastern Co-operative Group (ECOG) performance score 0–1; and (6) Child–Pugh class A or B. The exclusion criteria were as follows: (1) lenvatinib and sintilimab ≤2 cycles; (2) main portal vein completely blocked by tumor thrombus; (3) extrahepatic metastases; (4) other malignancies; (5) incomplete medical information; and (6) loss to follow-up.

### 2.2 Treatment

Four to six fiducial markers were implanted to determine the SBRT location using computed tomography (CT) simulation images (Accuray Inc., Sunnyvale, CA, USA). The planning target volume expanded the gross tumor volume (GTV) by 0.3–0.5 cm and avoided the organs at risk (OARs). The prescribed dose was 35–56 Gy/5–10 fractions. For dose constraints, the volume of liver receiving high dose radiation was minimized, particularly in cases with scattered intrahepatic satellite lesions. The tolerance doses of the OARs were determined according to the American Association of Physicists in Medicine TG-101 report (17).

Oral lenvatinib was administered within a week of the completion of SBRT at a dose of 8 mg (bodyweight ≤60 kg) or 12 mg (bodyweight >60 kg) once a day. Sintilimab (a programmed death-1 inhibitor) was administered intravenously on the first day of lenvatinib treatment at a dose of 200 mg every 3 weeks. Systemic therapy was not interrupted until disease progression, intolerable side effects, or serious AEs occurred.

TAE was performed according to a previously described method within 2 weeks before or after SBRT initiation (18) In brief, hepatic angiography was performed using a common femoral approach to locate the tumor, and 8–10 mL of lipiodol (Guerbet Pharmaceuticals, Paris, France) was slowly injected to embolize all vessels supplying the target tumor. TAE is commonly used in the following situations: (1) lesions protruding outward or into the liver capsule; (2) intratumoral hemorrhage, and (3) intrahepatic scattered lesions not covered by the target volume. TAE was not performed in the following cases: (1) main portal vein obstruction and high tumor burden, and the possibility of combination therapy leading to the deterioration of liver function; and (2) blood supply to the tumor being poor in the arterial phase of imaging and embolization being ineffective.

### 2.3 Assessment

The first follow-up was 6–8 weeks after SBRT, and subsequently, every 2–3 months until death or until December 2024. The follow-up included a physical examination, laboratory tests (such as routine blood tests, liver function, and serum tumor markers), and imaging (contrast-enhanced CT or magnetic resonance imaging of the abdomen and lung CT). The primary endpoints were progression-free survival (PFS) and OS; the secondary endpoint was local control (LC). PFS was defined as the duration from the date of SBRT initiation to the date of tumor progression or death from any cause. OS was defined as the time from SBRT initiation to death from any cause or the last follow-up. LC was defined as the duration between the initial date of SBRT and the date of irradiated tumor progression. Local tumor response was assessed using the modified Response Evaluation Criteria in Solid Tumors (19). ORR and disease control rate (DCR) were defined as the rates of complete response (CR) + partial response (PR) and CR + PR + stable disease, respectively. The tumor burden score (TBS) was calculated using the maximum diameter and number of intrahepatic tumors (20, 21). Treatment-related AEs (TRAEs) were graded according to the Common Terminology Criteria for Adverse Events (version 5.0). Additionally, patients were evaluated for radiation-induced liver disease (RILD) (22, 23).

### 2.4 Statistical analysis

Propensity score matching (PSM) analysis mitigated potential confounders and selection bias. The propensity score was estimated using a multivariate logistic regression model, and 1:1 matches between the two groups were performed using the nearest-neighbor method (caliper width=0.02). Variables included age, sex, virus infection, ECOG performance score, alpha-fetoprotein (AFP), Child–Pugh class, TBS, and PVTT type, and GTV includes only intrahepatic main lesion was considered.

Mann–Whitney–Wilcoxon test (continuous variables) and the chi-square test or Fisher’s exact test (discrete variables) were used to compare baseline characteristics, treatment responses, and TRAEs between the two groups. PFS, OS, and LC were analyzed using the Kaplan–Meier method and compared using the log-rank test and Cox regression model. All data were analyzed using R (version 4.0.3, Vienna, Austria) and Statistical Package for the Social Sciences (SPSS, version 25; IBM Corp., NY, US), and a p-value <0.05 indicated a statistically significant difference.

## 3 Results

### 3.1 Patient characteristics

**Figure 1:**
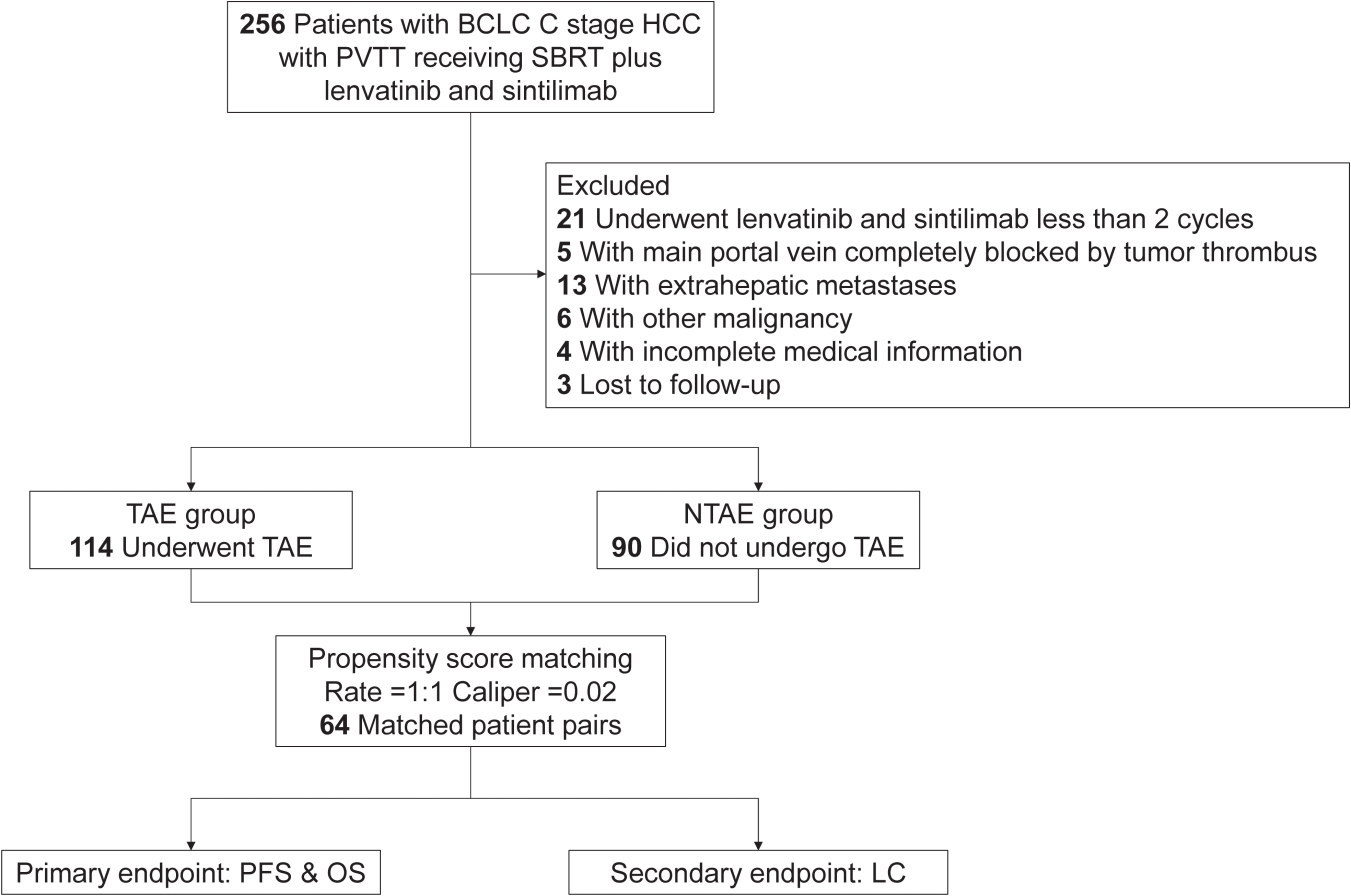
Patient flowchart. BCLC, Barcelona Clinic Liver Cancer; HCC, hepatocellular carcinoma; LC, local control; NTAE, non-transarterial embolization; OS, overall survival; PFS, progression-free survival; PVTT, portal vein tumor thrombus; SBRT, stereotactic body radiotherapy; TAE, transarterial embolization.

Between June 2018 and December 2022, 204 of 256 Barcelona Clinic Liver Cancer stage C patients with PVTT who were administered SBRT combined with lenvatinib and sintilimab met the inclusion criteria. Of these, 114 patients (55.9%) who underwent SBRT combined with lenvatinib, sintilimab, and TAE were assigned to the TAE group, while 90 patients (44.1%) treated with SBRT plus lenvatinib and sintilimab alone were included in the non-TAE (NTAE) group (**Figure 1**). The median ages were 57 years (range, 30–79 years) and 57 years (range, 30–81 years) in the TAE and NTAE groups, respectively. Male patients accounted for 90.4% (103/114) and 80.0% (72/90) of the TAE and NTAE groups, respectively. A TBS ≥8 was observed in 36.0% (41/114) and 45.6% (41/90) of patients in the TAE and NTAE groups, respectively. In the TAE group, PVTT types were classified as I (8.8% [10/114]), II (61.4% [70/114]), and III (29.8% [34/114]), and in 49.1% (56/114) of patients, the GTV was defined solely as the intrahepatic main lesion. In the NTAE group, the PVTT types included I (10.0% [9/90]), II (48.9% [44/90]), III (30.0% [27/90]), and IV (11.1% [10/90]), with 50.0% (45/90) targeting the intrahepatic main lesion alone. Of the 114 patients receiving TAE, the procedure preceded SBRT initiation in 42 (36.8%) and followed it in 72 (63.2%) patients, with all administrations adhering to the predefined 2-week peri-SBRT window. After PSM, the baseline characteristics were well-balanced between the two groups (**Table 1**).

**Table 1:** Baseline characteristics of the study cohort.

Characteristic	Before PSM			After PSM		
	TAE group (N=114)	NTAE group (N=90)	P	TAE group (N=64)	NTAE group (N=64)	P
Age, years (median, range)	57 (30-79)	57 (30-81)	0.351	57 (32-79)	56 (30-80)	0.540
Sex, n (%)			**0.036**			0.715
Male	103 (90.4)	72 (80.0)		59 (92.2)	61 (95.3)	
Female	11 (9.6)	18 (20.0)		5 (7.8)	3 (4.7)	
Virus infection, n (%)			0.902			0.496
Positive	110 (96.5)	88 (97.8)		62 (96.9)	64 (100.0)	
Negative	4 (3.5)	2 (2.2)		2 (3.1)	0	
ECOG performance, n (%)			0.894			0.157
0	102 (89.5)	80 (88.9)		54 (88.5)	60 (93.8)	
1	12 (10.5)	10 (11.1)		10 (15.6)	4 (6.3)	
AFP, ng/mL, n (%)			0.159			0.596
<400	52 (45.6)	50 (55.6)		31 (48.4)	34 (53.1)	
≥400	62 (54.4)	40 (44.4)		33 (51.6)	30 (46.9)	
Child-Pugh class, n (%)			0.594			0.230
A	89 (78.1)	73 (81.1)		44 (68.8)	50 (78.1)	
B	25 (21.9)	17 (18.9)		20 (31.2)	14 (21.9)	
TBS, n (%)			0.165			0.722
<8	73 (64.0)	49 (54.4)		35 (54.7)	37 (57.8)	
≥8	41 (36.0)	41 (45.6)		29 (45.3)	27 (42.2)	
Tumor size, cm, n (%)			0.264			0.717
<8	77 (67.5)	54 (60.0)		38 (59.4)	40 (62.5)	
≥8	37 (32.5)	36 (40.0)		26 (40.6)	24 (37.5)	
Tumor number, n (%)			0.818			0.824
<3	23 (20.2)	17 (49.3)		12 (18.8)	13 (20.3)	
≥3	91 (79.8)	73 (50.7)		52 (81.2)	51 (79.7)	
PVTT type, n (%)			**0.001**			0.078
I	10 (8.8)	9 (10.0)		5 (7.8)	5 (7.8)	
II	70 (61.4)	44 (48.9)		38 (59.4)	31 (48.4)	
III	34 (29.8)	27 (30.0)		21 (32.8)	22 (34.4)	
IV	0	10 (11.1)		0	6 (9.4)	
GTV includes only intrahepatic main lesions #, n (%)			0.901			1.000
Absent	58 (50.9)	45 (50.0)		30 (46.9)	30 (46.9)	
Present	56 (49.1)	45 (50.0)		34 (53.1)	34 (53.1)	

#Intrahepatic main lesions include PVTT and the adjacent parenchymal tumors.

AFP, alpha-fetoprotein; ECOG, Eastern Cooperative Oncology Group; GTV, gross tumor volume; NTAE, non-transarterial embolization; PSM, propensity score matching; PVTT, portal vein tumor thrombus; TAE, transarterial embolization; TBS, tumor burden score.

Bold indicates statistical significance.

### 3.2 Efficacy outcomes

The ORR and DCR remained consistent before and after PSM (pre-PSM vs. post-PSM: TAE group ORR, 46.5% vs. 40.6%; DCR, 83.3% vs. 79.7%; NTAE group ORR, 42.2% vs. 39.1%; DCR, 79.7% vs. 81.3%). In the post-PSM cohort, CR was achieved in 16.4% (10/64) and PR in 25.0% (16/64) of the patients in the TAE group, compared to 6.2% (4/64) CR and 32.8% (21/64) PR in the NTAE group. No significant differences were observed in the ORR (40.6% [95% CI: 28.3–53.0%] vs. 39.1% [26.8–51.3%], p=0.861) or DCR (79.7% [69.6–89.8%] vs. 81.3% [71.4–91.1%], p=0.819) between the two groups (**Table 2**).

**Table 2:** Therapeutic efficacy.

Therapeutic response assessment	Before PSM			After PSM		
	TAE group (N=114)	NTAE group (N=90)	P	TAE group (N=64)	NTAE group (N=64)	P
ORR, n (%; 95% CI)	53 (46.5; 37.2-55.8)	38 (42.2; 31.8-52.6)	0.542	26 (40.6; 28.3-53.0)	25 (39.1; 26.8-51.3)	0.857
CR, n (%)	14 (12.3)	6 (6.7)	0.181	10 (16.4)	4 (6.2)	0.157
PR, n (%)	39 (34.2)	32 (35.6)	0.841	16 (25.0)	21 (32.8)	0.330
SD, n (%)	42 (36.8)	39 (43.3)	0.347	25 (39.1)	27 (42.2)	0.719
PD, n (%)	19 (16.7)	13 (14.4)	0.665	13 (20.3)	12 (18.8)	0.824
DCR, n (%; 95% CI)	95 (83.3; 76.4-90.3)	77 (85.6; 78.2-93.0)	0.665	51 (79.7; 69.6-89.8)	52 (81.3; 71.4-91.1)	0.824

CI, confidence interval; CR, complete response; DCR, disease control rate; NTAE, non-transarterial embolization; ORR, objective response rate; PD, progressive disease; PR, partial response; PSM, propensity score matching; SD, stable disease; TAE, transarterial embolization.

**Figure 2:**
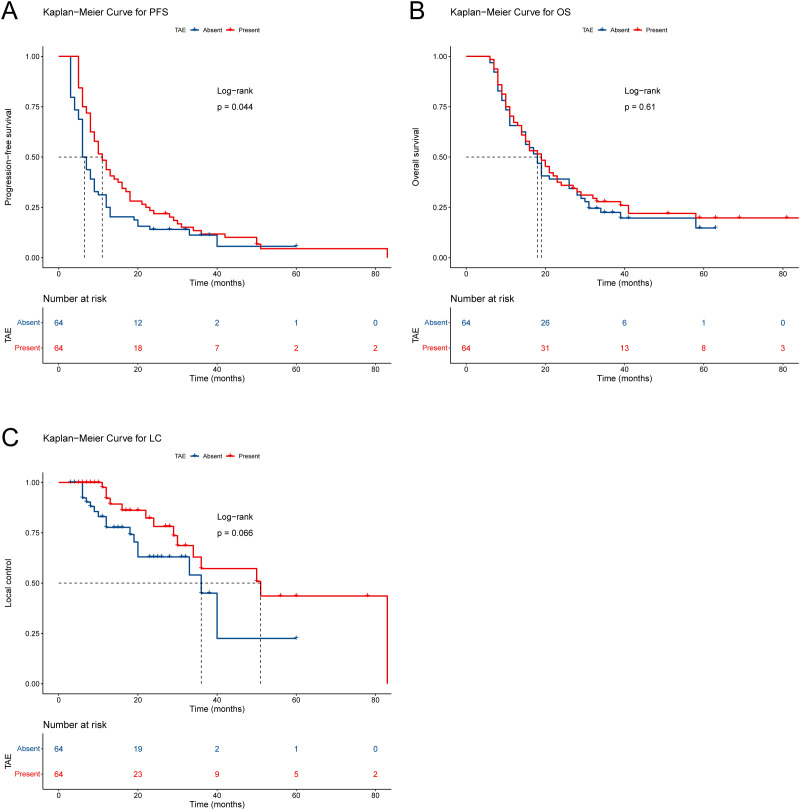
Survival analysis. Kaplan−Meier curves for **(A)** progression-free survival, **(B)** overall survival, and **(C)** local control. LC, local control; OS, overall survival; PFS, progression-free survival; TAE, transarterial embolization.

At the cutoff (December 2024), the median follow-up duration was 59.0 months (95% CI: 39.8–78.2 months; TAE group: 63.0 months [59.8–66.2]; NTAE group: 39.0 months [34.7–43.3]). Disease progression or death occurred in 96.9% (62/64) and 89.1% (57/64) of patients who underwent TAE and NTAE, respectively. The TAE group demonstrated a significantly longer median progression-free survival (mPFS) than the NTAE group (11.0 months [95% CI: 8.4–13.6] vs. 6.0 months [5.0–7.0]; hazard ratio [HR]=0.71, p=0.044), corresponding to a 29% reduction in progression risk (**Figure 2a**). Median overall survival (mOS) was comparable between the groups (TAE: 19.0 months [14.3–23.7]; NTAE: 18.0 months [14.9–21.1]; HR = 0.90, p=0.606; **Figure 2b**). The TAE group exhibited a trend toward improved median local control (mLC) (51.0 months [25.7–76.3] vs 36.0 months [24.1–47.9]; HR = 0.54, p=0.066; **Figure 2c**). The 1-, 2-, and 3-year LC rates were 56.3%, 31.3%, and 17.2%, respectively, in the TAE group, and 48.4%, 23.4%, and 9.4%, respectively, in the NTAE group.

Multivariate analysis (MVA) identified the addition of TAE as an independent prognostic factor for prolonged PFS (HR = 0.52 [95% CI: 0.36–0.76]) and LC (HR = 0.34 [0.16–0.74]). Radiation targeting solely intrahepatic main lesions independently increased risks of disease progression (HR = 1.73 [1.19–2.52]), mortality (HR = 1.62 [1.08–2.44]), and local failure (HR = 2.47 [1.13–5.41]). AFP ≥400 ng/mL was associated with worse OS (HR = 1.73 [1.13–2.64]) and LC (HR = 2.33 [1.06–5.11]) (**Table 3**).

**Table 3:** Univariate and multivariate analysis of progression-free survival, local control, and overall survival.

For matched groups (N=64)	Progression-free survival				Overall survival			
	UVA		MVA		UVA		MVA	
	HR (95% CI)	P	HR (95% CI)	P	HR (95% CI)	P	HR (95% CI)	P
Group (NTAE vs. TAE)	0.68 (0.47-0.98)	**0.04**	0.52 (0.36-0.76)	**<0.001**	0.90 (0.61-1.33)	0.592		
Sex (Female vs. Male)	1.63 (0.79-3.39)	0.187			2.08 (0.84-5.11)	0.112		
Age, years (<60 vs. ≥60)	1.22 (0.84-1.77)	0.304			1.21 (0.81-1.81)	0.344		
Child-Pugh class (A vs. B)	1.38 (0.91-2.08)	0.127			2.07 (1.35-3.19)	**0.001**	1.56 (0.99-2.47)	0.058
ECOG (0 vs. 1)	1.12 (0.64-1.98)	0.687			0.98 (0.52-1.84)	0.95		
PVTT type (I vs. II&III&IV)	1.41 (0.71-2.80)	0.322			1.83 (0.80-4.18)	0.152		
Tumor size, cm (<8 vs. ≥8)	1.81 (1.23-2.66)	**0.002**	1.86 (0.77-4.50)	0.168	1.84 (1.23-2.75)	**0.003**	1.14 (0.46-2.81)	0.774
Tumor number (<3 vs. ≥3)	1.79 (1.12-2.87)	**0.015**	0.73 (0.41-1.30)	0.282	3.23 (1.79-5.82)	**<0.001**	1.18 (0.59-2.37)	0.646
TBS (<8 vs. ≥8)	2.00 (1.37-2.93)	**<0.001**	0.89 (0.37-2.15)	0.795	2.16 (1.45-3.23)	**<0.001**	1.26 (0.50-3.18)	0.618
Virus infection (Negative vs. Positive)	1.13 (0.28-4.62)	0.86			2.71 (0.38-19.46)	0.322		
AFP, ng/ml (<400 vs. ≥400)	1.27 (0.88-1.82)	0.202			1.50 (1.01-2.23)	**0.042**	1.78 (1.15-2.78)	**0.011**
GTV includes only intrahepatic main lesions (absent vs. present)	7.94 (4.78-13.18)	**<0.001**	10.86 (5.92-19.93)	**<0.001**	8.65 (5.36-13.98)	**<0.001**	9.09 (5.07-16.3)	**<0.001**
For matched groups (N=64)	Local control							
	UVA				MVA			
	HR (95% CI)	P			HR (95% CI)	P		
Group (NTAE vs. TAE)	0.50 (0.24-1.06)	**0.07**			0.34 (0.16-0.74)	**0.007**		
Sex (Female vs. Male)	1.63 (0.47-5.58)	0.439						
Age, years (<60 vs. ≥60)	1.00 (0.47-2.15)	0.998						
Child-Pugh class (A vs. B)	0.34 (0.08-1.42)	0.139						
ECOG (0 vs. 1)	0.48 (0.14-1.64)	0.243						
PVTT type (I vs. II&III&IV)	2.19 (0.52-9.31)	0.287						
Tumor size, cm (<8 vs. ≥8)	1.17 (0.48-2.86)	0.728						
Tumor number (<3 vs. ≥3)	2.11 (0.90-4.98)	**0.087**			1.47 (0.57-3.77)	0.421		
TBS (<8 vs. ≥8)	1.32 (0.56-3.15)	0.885						
Virus infection (Negative vs. Positive)	NE (0-NE)	0.997						
AFP, ng/ml (<400 vs. ≥400)	1.83 (0.90-3.72)	**0.092**			2.30 (1.11-4.77)	**0.025**		
GTV includes only intrahepatic main lesions (absent vs. present)	7.88 (2.74-22.66)	**<0.001**			11.29 (3.37-37.81)	**<0.001**		

Any factors that were statistically significant at P <10% in the univariate analysis were candidates for entry into a multivariable Cox analysis.

AFP, alpha-fetoprotein; CI, confidence interval; ECOG, Eastern Cooperative Oncology Group; GTV, gross tumor volume; HR, hazard ratio; MVA, multivariate analysis; NTAE, non-transarterial embolization; PVTT, portal vein tumor thrombus; RT, radiotherapy; TAE, transarterial embolization; TBS, tumor burden score; UVA, univariate analysis.

Bold indicates statistical significance.

### 3.3 Safety outcomes

The TRAEs are summarized in **Table 4**. We analyzed both any grade and grade ≥3 TRAEs to compare the safety of SBRT combined with lenvatinib and sintilimab with that without TAE. No treatment-related death occurred during the study period. Grade ≥1 TRAEs were reported in 96.9% (124/128) patients. The most common TRAEs in the TAE group were decreased white blood cell count (46.9%), decreased lymphocyte count (34.4%), and rash (32.9%), whereas patients in the NTAE group frequently experienced rashes (48.5%), decreased lymphocyte count (42.2%), and lowered white blood cell count (39.1%). No significant differences were observed in the incidence of TRAEs of any grade or type between the two groups. Grade ≥3 TRAEs occurred in 32 patients (50.0%) in the TAE and NTAE groups. The addition of TAE did not increase the overall incidence of TRAEs. RILD occurred in 12 (18.8%) and 11 patients (17.2%) in the TAE and NTAE groups, respectively.

**Table 4:** Safety summary.

Treatment-related adverse events	TAE group (N=64)		NTAE group (N=64)		P		
	Any grade	Grade 3-4	Any grade	Grade 3-4	Any grade	Grade 3-4	
	n (%)		n (%)				
Rash	20 (31.3)	1 (1.6)	30 (46.9)	1 (1.6)	0.070	1.000	
Constipation	14 (21.9)	1 (1.6)	14 (21.9)	0	1.000	1.000	
Palmar-plantar erythrodysesthesia syndrome	13 (20.3)	0	15 (23.4)	1 (1.6)	0.669	1.000	
Anorexia	18 (28.1)	0	13 (20.3)	0	0.320	–	
Nausea	14 (21.9)	0	6 (9.4)	0	0.051	–	
Malaise	10 (15.6)	1 (1.6)	7 (10.9)	0	0.435	1.000	
Diarrhea	9 (14.1)	0	8 (12.5)	0	0.795	–	
Hypertension	4 (6.3)	1 (1.6)	8 (12.5)	0	0.363	1.000	
Fever	6 (9.4)	1 (1.6)	5 (7.8)	0	0.752	1.000	
Abdominal pain	3 (4.7)	0	5 (7.8)	0	0.715	–	
Proteinuria	2 (3.1)	0	2 (3.1)	0	1.000	–	
White blood cell decreased	26 (40.6)	4 (6.3)	21 (32.8)	4 (6.3)	0.359	1.000	
Neutrophil count decreased	15 (23.4)	2 (3.1)	10 (15.6)	0	0.265	0.496	
Lymphocyte count decreased	9 (14.1)	13 (20.3)	12 (18.8)	15 (23.4)	0.474	0.669	
Platelet count decreased	10 (15.6)	3 (4.7)	17 (26.6)	4 (6.3)	0.129	0.697	
Blood bilirubin increased	0	3 (4.7)	0	3 (4.7)	–	1.000	
ALT/AST increased	2 (3.1)	2 (3.1)	2 (3.1)	4 (6.3)	1.000	0.676	
ALP increased	0	0	1 (1.6)	0	1.000	–	

ALP, alkaline phosphatase; ALT, alanine aminotransferase; AST, aspartate aminotransferase; NTAE, non-transarterial embolization; TAE, transarterial embolization.

## 4 Discussion

PVTT is an independent predictor of poor prognosis in HCC, with supportive care alone resulting in a median survival of less than 6 months (24, 25). Although systemic therapies and LRT have demonstrated efficacy in patients with HCC and PVTT, an optimal therapeutic strategy remains undefined (14, 26). This is the first clinical cohort study to compare the efficacy and safety of SBRT combined with targeted immunotherapy (lenvatinib + sintilimab), with and without TAE. Our results indicated that adding TAE significantly prolonged the mPFS (11.0 vs 6.0 months, p=0.044) and showed a trend toward improved OS and LC. These findings suggest the potential therapeutic options for patients with HCC and PVTT.

Previous studies reported mPFS of 4.6–9.6 months in patients with PVTT treated with radiotherapy plus targeted immunotherapy, consistent with the outcomes observed in our NTAE group (4, 24). The addition of TAE reduced the risk of disease progression by 48%, attributable to the synergistic effects of dual LRT. The significant PFS benefit observed with the addition of TAE underscores the synergistic potential of combining dual locoregional therapy with systemic agents. This rationale for intensifying local therapy in combination with systemic regimens is corroborated by emerging real-world evidence (3, 27). Notably, a recent nationwide target trial emulation study demonstrated that in patients with advanced HCC and Vp4-type PVTT, augmenting systemic immunotherapy and targeted therapy with aggressive local interventional modalities significantly improved PFS compared to systemic treatment alone (28). This benefit was accompanied by a comparable pattern of first disease progression between the groups (**Supplementary Table S1**), suggesting a broad delay across progression patterns rather than a shift in failure modes. However, despite the improved PFS, the mOS in the TAE group (19.0 months) did not significantly differ from that in the NTAE group (18.0 months). Subsequent therapies after the initial treatment may confound survival outcomes, leading to a lack of OS benefits from PFS improvements. In addition, preclinical evidence suggests that TAE induces an immunosuppressive microenvironment that is insufficient to maximize immune checkpoint inhibitor responses, potentially explaining the lack of OS benefit (25).

A meta-analysis by Xiao-fei et al. demonstrated that SBRT combined with transcatheter arterial chemoembolization (TACE) improved ORR compared with TACE alone in patients with PVTT but showed no significant advantage over SBRT monotherapy (14). Similarly, our study found no significant improvement in ORR or DCR with dual LRT (TAE + SBRT) compared with SBRT alone (40.6% vs. 39.1%, p=0.861). This may be attributed to the vascular heterogeneity between PVTT and parenchymal tumors, as SBRT inherently targets both lesion types (26).

Ting-Shi et al. reported a trend toward improved 1- and 2-year LC rates with radiotherapy combined with TACE versus radiotherapy alone (41.5% vs. 17.1% and 24.4% vs. 9.8%, respectively; P >0.05) in patients with HCC showing macrovascular invasion, aligning with the LC outcomes in our TAE group (29). Furthermore, MVA demonstrated that adding TAE was an independent prognostic factor for disease progression, significantly reducing the risk of disease progression and improving LC. Achieving adequate local control in large tumors is challenging due to difficulties in obtaining sufficient ablative margins and the frequent presence of microvascular invasion and peritumoral satellite lesions (30). This biological rationale supports a spatially complementary strategy, wherein SBRT delivers focused ablation to the main tumor volume, while TAE addresses the peripheral target volume and potential satellite lesions. Similarly, Tiziana et al. reviewed 40 patients with unresectable HCC treated with SBRT plus TACE or TACE alone and revealed superior 1-year LC rates in the SBRT cohort (84% vs. 23%) (31). These findings underscore the potential of dual LRT for intrahepatic disease control and warrant prospective studies to optimize LRT sequencing and combination strategies.

Studies on combining SBRT with lenvatinib, sintilimab, and TAE are limited, and their safety profiles remain poorly defined. The selection of TAE over TACE was primarily informed by safety considerations, aiming to avoid additive hepatotoxicity on top of the established profiles of lenvatinib and sintilimab. This safety-oriented premise was validated by the clinical outcomes: the addition of dual LRT did not significantly increase the incidence of systemic therapy-related AE, such as rash and hand-foot syndrome, compared with the NTAE group. Hematologic and hepatic toxicities were comparable between the two groups, and the incorporation of TAE did not exacerbate these toxicities. These findings suggest that SBRT combined with lenvatinib, sintilimab, and TAE is a feasible and well-tolerated treatment option for patients with advanced HCC and PVTT.

Given the retrospective design of our study, the timing of TAE relative to SBRT was determined by real-world clinical practice. Consequently, the potential impact of specific treatment sequencing on immunologic or clinical efficacy could not be assessed and merits future investigation. Additionally, the study period coincided with the COVID-19 pandemic, which may have introduced biases during follow-up and reexamination. Long-term follow-up for late toxicities and subsequent therapies would be valuable. Furthermore, biomarkers indicating the response and prognosis of patients with PVTT after dual LRT, such as the immune profiling and circulating tumor DNA, also need further study. Large-scale prospective studies are required to substantiate these results.

## 5 Conclusion

In conclusion, this real-world study suggests that adding TAE to SBRT plus lenvatinib and sintilimab may significantly improve PFS in patients with HCC with PVTT without increasing severe toxicities. However, the lack of OS benefits and the retrospective design warrant further validation in larger prospective cohorts.

## Data Availability

The original contributions presented in the study are included in the article/**Supplementary Material**. Further inquiries can be directed to the corresponding authors.
