# Supplementary material for: Stereotactic body radiotherapy plus lenvatinib and sintilimab with and without transarterial embolization for advanced hepatocellular carcinoma with portal vein tumor thrombus: a dual-center, propensity score-matched retrospective analysis
Source: Front Immunol. 2026 Mar 4;17:1644027. doi: 10.3389/fimmu.2026.1644027 (PMC12996245; doi:10.3389/fimmu.2026.1644027)
Supplement: Supplementary file 1 [file DataSheet1.docx]

Table S1. Patterns of disease progression

| Patterns of disease progression | | Before PSM | | | | | After PSM | | | | | |
| --- | --- | --- | --- | --- | --- | --- | --- | --- | --- | --- | --- | --- |
|  |  | TAE group (N=114) | NTAE group (N=90) | P | | | TAE group (N=64) | NTAE group (N=64) | | P | | |
| GTV, n (%) | 37 (32.5) | | 27 (30.0) | | 0.707 | 19 (29.7) | | | 18 (28.1) | | 0.845 |  |
| Intrahepatic lesion, n (%) | 41 (36.0) | | 35 (38.9) | | 0.668 | 24 (37.5) | | | 25 (39.1) | | 0.856 |  |
| Extrahepatic lesion, n (%) | 35 (30.7) | | 22 (24.4) | | 0.323 | 18 (28.1) | | | 16 (25.0) | | 0.689 |  |
| Free from progression, n (%) | 6 (5.3) | | 9 (10.0) | | 0.198 | 4 (6.3) | | | 7 (10.9) | | 0.344 |  |

Abbreviations: GTV, gross tumor volume; NTAE, non-transarterial embolization; PSM, propensity score matching; TAE, transarterial embolization.
